# Palmitic Acid Induces Müller Cell Inflammation that is Potentiated by Co-treatment with Glucose

**DOI:** 10.1038/s41598-018-23601-1

**Published:** 2018-04-03

**Authors:** Megan E. Capozzi, Meredith J. Giblin, John S. Penn

**Affiliations:** 1 Department of Molecular Physiology and Biophysics at Vanderbilt University, 1301 Medical Center Drive TVC B706-A, Nashville, TN 37232-0028 USA; 2 Department of Cell and Developmental Biology at Vanderbilt University, 1301 Medical Center Drive TVC B706-A, Nashville, TN 37232-0028 USA; 3 Department of Ophthalmology and Visual Sciences at Vanderbilt University Medical Center, 1301 Medical Center Drive TVC B706-A, Nashville, TN 37232-0028 USA

## Abstract

Chronic hyperglycemia is thought to be the major stimulator of retinal dysfunction in diabetic retinopathy (DR). Thus, many diabetes-related systemic factors have been overlooked as inducers of DR pathology. Cell culture models of retinal cell types are frequently used to mechanistically study DR, but appropriate stimulators of DR-like factors are difficult to identify. Furthermore, elevated glucose, a gold standard for cell culture treatments, yields little to no response from many primary human retinal cells. Thus, the goal of this project was to demonstrate the effectiveness of the free fatty acid, palmitic acid and compare its use alone and in combination with elevated glucose as a stimulus for human Müller cells, a retinal glial cell type that is activated early in DR pathogenesis and uniquely responsive to fatty acids. Using RNA sequencing, we identified a variety of DR-relevant pathways, including NFκB signaling and inflammation, intracellular lipid signaling, angiogenesis, and MAPK signaling, that were stimulated by palmitic acid, while elevated glucose alone did not significantly alter any diabetes-relevant pathways. Co-treatment of high glucose with palmitic acid potentiated the expression of several DR-relevant angiogenic and inflammatory targets, including *PTGS2* (COX-2) and *CXCL8* (IL-8).

## Introduction

Based on the results of several clinical trials demonstrating that tight glycemic control slows the progression of DR, including the Diabetes Control and Complications Trial (DCCT) and U.K. Prospective Diabetes Study (UKPDS), hyperglycemia has been considered the driving cause of DR pathology^1,2^. Owing to the long recognized reputation for glucose in DR pathogenesis, basic research has focused on elevated glucose in cell culture models to recapitulate mechanisms of DR. Yet, results from several clinical studies suggest glucose may not be the primary driver of DR, because overt hyperglycemia is not necessary for the development of pathology. For example, multiple case studies observed DR pathology in patients with relatively normal glucose tolerance^3–5^. Additional evidence comes from case studies of patients with bariatric surgery, in which there have been instances when retinopathy progressed, despite lowering of HbA1c^6^. This provides the impetus to develop an understanding of non-glucose driven pathology in both *in vitro* and *in vivo* disease models for mechanistic understanding of DR pathogenesis.

Recent studies have demonstrated a strong association between dyslipidemia and DR. In the Fenofibrate Intervention and Event Lowering in Diabetes (FIELD) and The Action to Control Cardiovascular Risk in Diabetes (ACCORD) studies, the lipid-lowering drug, fenofibrate, delayed retinopathy progression, independent of glycemic control^7,8^. In humans and animal models, diabetes increases fatty acid concentrations in systemic circulation and tissues, leading to inflammation, insulin resistance, and disease progression^9,10^. Mounting evidence supports the use of fatty acids as a diabetes-relevant stimulus in non-ocular experimental contexts, but their use in the context of DR remains limited^11–14^. However, there is evidence that these fatty acids evoke inflammatory responses in retinal microvascular endothelial cells^11,14^. Serum profiles from diabetic patients and retinal tissue profiles from experimental models of diabetes demonstrate that one saturated fatty acid, palmitic acid (PA), is elevated above others^12,15,16^. These data suggest that elevated fatty acids, and particularly PA, may be causally linked to retinal inflammation occurring early in the pathogenesis of DR.

It is important to note that dyslipidemia occurs in the absence of diabetes, and hyperlipidemic patients do not have the same retinal pathology as that observed in DR. This provides the impetus to assess the combination of diabetes-relevant metabolic changes for the design of DR-relevant cell culture conditions. Thus, while previous work in the field has demonstrated limited effects of glucose in cell cultures that are independent of osmolarity^13,17^, there still may be a benefit to its use in combination with free fatty acids. Moreover, our previous studies revealed that retinal Müller cells were uniquely responsive to fatty acid stimulation when compared to other retinal cell types involved in DR pathology^13^. Notably, Müller cells are also highly responsive to metabolic alterations in the retina, and their activation is one of the earliest changes observed in DR^18,19^.

The goal of the present study was to compare PA- and D-glucose-treated primary human Müller cell cultures and to determine whether combination treatment further promoted DR-relevant pathways using whole transcriptome analysis for differential gene expression. We first demonstrate the effects of each stimulus (palmitic acid and high glucose) individually, as whole transcriptome analysis of primary human Müller cells under these culture conditions has not been reported. We next describe whole transcriptome analysis from co-treated Müller cell cultures and confirmation of the results from these analyses, to determine whether hyperglycemic- and hyperlipidemic-mimicking culture conditions synergize to elicit DR pathogenic responses. The platform described herein will provide a basis for both mechanistic studies as well as assessment of therapeutic strategies for DR using *in vitro* culture models.

## Results

### RNAseq Quality and Alignment

In order to determine the effects of PA, D-glucose, and their combination on transcriptional changes in Müller cells, RNAseq was performed. The four experimental groups (2 samples of each) were treated as follows: LG/BSA (24 hours of L-glucose, followed by 24 hours of L-glucose plus BSA); LG/PA (24 hours of L-glucose, followed by 24 hours of L-glucose plus PA); DG/BSA (24 hours of D-glucose, followed by 24 hours of D-glucose plus BSA); DG/PA (24 hours of D-glucose, followed by 24 hours of D-glucose plus PA). As shown in Supplementary Fig. 1, Müller cell identity was verified in all treatment groups by immunocytochemical staining of glial fibrillary activated protein (GFAP) and glutamine synthase (GS). The average read count was 11318908.38 reads per sample, mapping on average 93.88% of the human genome (Table 1). The read counts were not statistically different between treatment groups using an ANOVA (p = 0.2387).

**Table 1:** Summary of reads mapping to the human genome using QC3.

Sample	Total Reads	Mapped (%)
LG/BSA1	11058763	93.38
LG/BSA2	10249362	93.83
LG/PA1	11245915	93.79
LG/PA2	12642057	93.71
DG/BSA1	11138539	94.01
DG/BSA2	10804232	93.25
DG/PA1	11808323	94.78
DG/PA1	11604076	94.31
Average	11318908.38	93.88

### The Effect of PA Treatment on Human Müller Cell Gene Expression

As shown in Supplementary Fig. 2, 2540 hits were significantly changed as demonstrated by all three statistical tests (DESeq. 2, edgeR, and baySeq) between the LG/BSA and LG/PA groups. Of these, 740 were down-regulated while 1800 were up-regulated. The protein-coding genes with the largest log2fold changes are reported in Table 2.

**Table 2:** List of top 15 up-regulated genes (bold) and down-regulated (italic) genes in comparison of LG/BSA vs. LG/PA samples.

Ensembl Gene ID	Gene Target	Log2fold change	Adj p value
**ENSG00000081041**	**CXCL2**	**5.131169228**	**3.48E-121**
**ENSG00000180535**	**BHLHA15**	**4.970842312**	**2.06E-107**
**ENSG00000167861**	**HID1**	**4.556667723**	**4.04E-110**
**ENSG00000078081**	**LAMP3**	**4.125085146**	**3.52E-55**
**ENSG00000173110**	**HSPA6**	**4.097445282**	**6.88E-64**
**ENSG00000167772**	**ANGPTL4**	**3.943015061**	**0**
**ENSG00000004799**	**PDK4**	**3.804721573**	**1.73E-73**
**ENSG00000163734**	**CXCL3**	**3.763486125**	**9.79E-57**
**ENSG00000130487**	**KLHDC7B**	**3.660859835**	**4.54E-51**
**ENSG00000162772**	**ATF3**	**3.640394145**	**0**
**ENSG00000169429**	**CXCL8**	**3.546291509**	**4.45E-97**
**ENSG00000147872**	**PLIN2**	**3.366381211**	**7.28E-273**
**ENSG00000137491**	**SLCO2B1**	**3.359650884**	**1.19E-23**
**ENSG00000099958**	**DERL3**	**2.985336774**	**9.33E-42**
**ENSG00000133134**	**BEX2**	**2.952925525**	**4.57E-46**
*ENSG00000180660*	*MAB21L1*	*−1.477045754*	*5.61E-19*
*ENSG00000186847*	*KRT14*	*−1.497222046*	*7.37E-102*
*ENSG00000167244*	*IGF2*	*−1.501273659*	*7.04E-114*
*ENSG00000131095*	*GFAP*	*−1.525215644*	*2.41E-13*
*ENSG00000205221*	*VIT*	*−1.564053796*	*1.28E-28*
*ENSG00000105989*	*WNT2*	*−1.597706963*	*3.55E-46*
*ENSG00000130592*	*LSP1*	*−1.628532041*	*4.50E-10*
*ENSG00000137672*	*TRPC6*	*−1.629488495*	*3.81E-45*
*ENSG00000179772*	*FOXS1*	*−1.669083989*	*1.64E-13*
*ENSG00000163909*	*HEYL*	*−1.703173713*	*3.48E-08*
*ENSG00000104415*	*WISP1*	*−1.752999127*	*9.97E-21*
*ENSG00000185585*	*OLFML2A*	*−1.826404845*	*7.21E-51*
*ENSG00000078401*	*EDN1*	*−1.96860795*	*5.77E-41*
*ENSG00000163815*	*CLEC3B*	*−2.078011452*	*3.05E-43*
*ENSG00000106511*	*MEOX2*	*−2.333794203*	*2.36E-23*

Log2fold change is the average from all statistical tests. Adjusted p-value is reported from EdgeR analysis.

**Figure 1:**
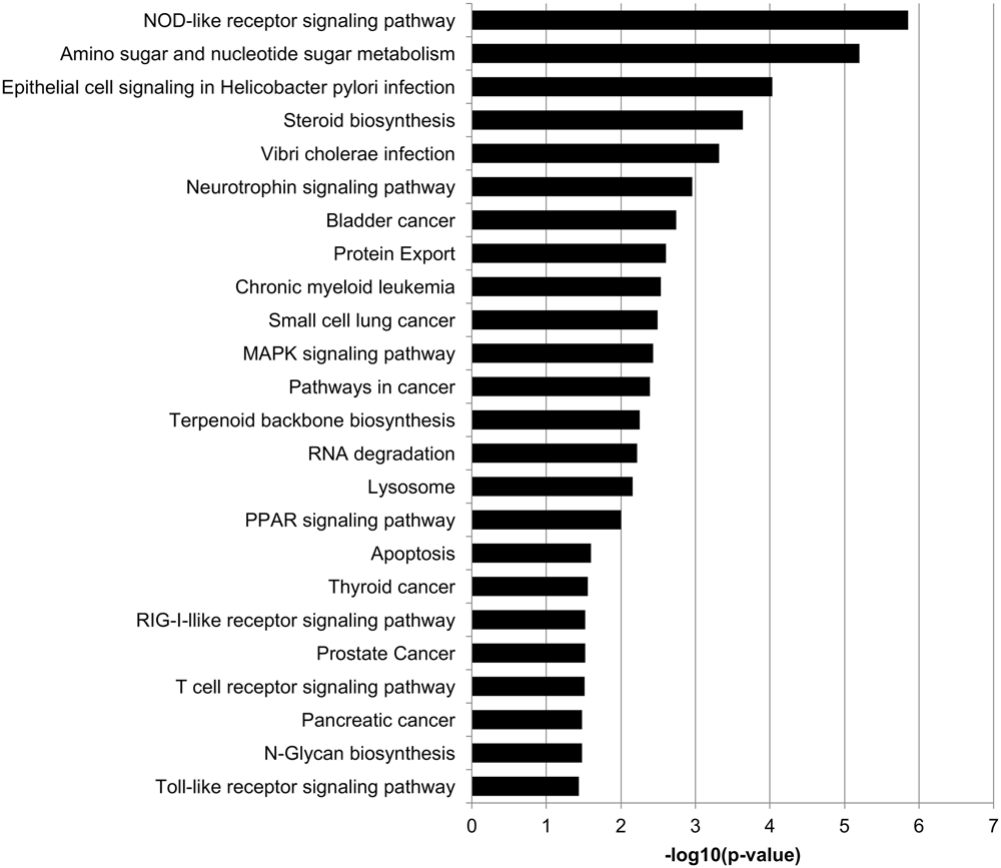
KEGG pathways enriched by PA-treatment alone. Pathway enrichment was determined using DAVID v6.7 with a p < 0.05. Twenty-four pathways were significantly up-regulated in response to PA treatment, compared to BSA control.

**Figure 2:**
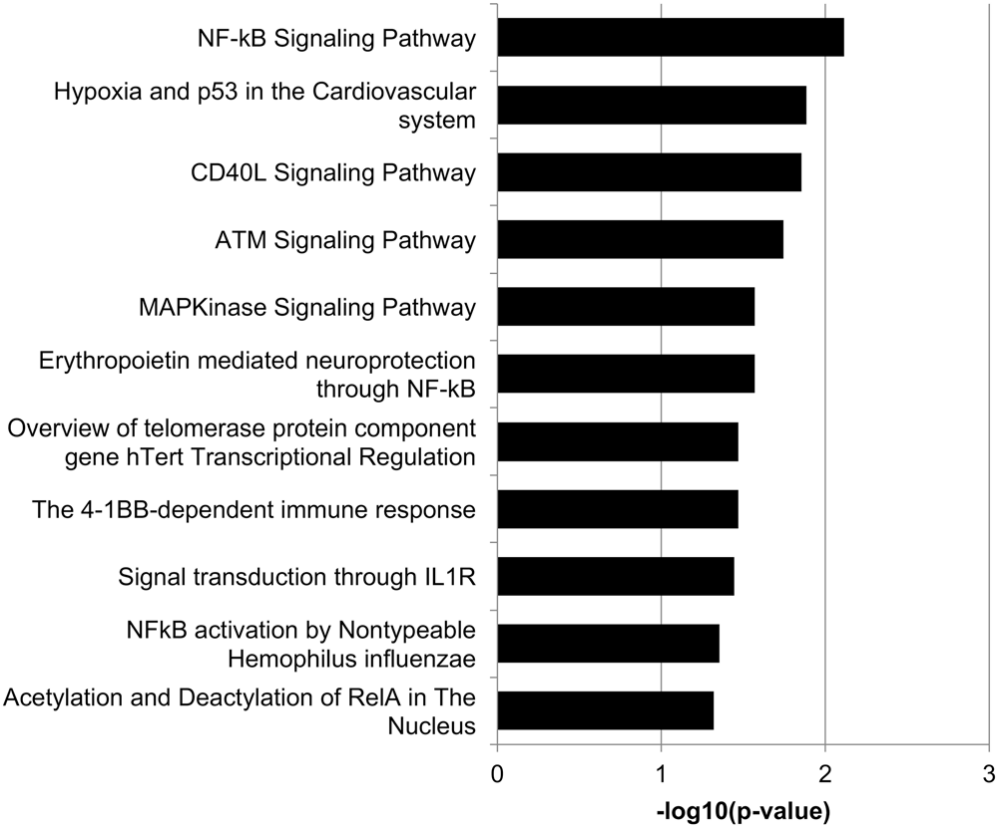
Biocarta pathways enriched by PA-treatment alone. Pathway enrichment was determined using DAVID v6.7 with a p < 0.05. Eleven pathways were significantly up-regulated in response to PA treatment, compared to BSA control.

Pathway analysis was performed using the significantly up-regulated genes to determine which biological pathways were enriched in our data set. Of these genes, 582 were identified in the KEGG database (Fig. 1) and 173 were identified in the Biocarta database (Fig. 2). Twenty-four pathways were significantly enriched by KEGG analysis and 11 pathways were significantly enriched by Biocarta analysis. Many interesting and relevant pathways were enriched, including inflammatory pathways linked to “NOD-like receptor signaling pathway” (19 hits) “RIG-I-like receptor signaling” (12 hits) and “Toll-like receptor signaling pathway” (15 hits) by KEGG analysis. Additionally, in Biocarta pathway analysis, the “NFκB Signaling Pathway” (8 hits) and several associated NFκB pathway terms were enriched, in addition to “Signal transduction through IL1R” (8 hits). Additional DR-relevant pathways in the KEGG analysis include “Amino sugar and nucleotide sugar metabolism” (15 hits) “Neurotrophin signaling pathway” (22 hits), “MAPK signaling pathway” (36 hits), “PPAR signaling pathway” (13 hits), and “Apoptosis” (7 hits). MAPK signaling was also significantly enriched in Biocarta analysis.

### The Effect of D-glucose Treatment on Müller Cell Gene Expression

Using the stringency of all three statistical tests, only four genes were significantly altered between the LG/BSA and DG/BSA groups. Therefore, the 74 genes that were significantly altered according to any two statistical tests were used in pathway analysis in order to have a large enough population of hits. The top ten up-regulated and down-regulated hits from this group are reported in Table 3. Using this gene list, only the “Splicesome” pathway was significantly altered by KEGG analysis (6 hits; p = 4.2E-5).

**Table 3:** List of top 10 up-regulated genes (bold) and down-regulated (italic) genes in comparison of LG/BSA vs. DG/BSA samples.

Ensembl Gene ID	Gene Target	Log2fold change	Adj p value
**ENSG00000134321**	**RSAD2**	**4.923508774**	**4.09E-08**
**ENSG00000269483**	**AC006272.1**	**2.919487587**	**0.019466295**
**ENSG00000164344**	**KLKB1**	**2.548189429**	**0.00071155**
**ENSG00000111335**	**OAS2**	**2.17216683**	**7.02E-05**
**ENSG00000089127**	**OAS1**	**2.168790961**	**4.62E-06**
**ENSG00000183486**	**MX2**	**2.010356923**	**0.000545912**
**ENSG00000137959**	**IFI44L**	**1.925914084**	**6.37E-08**
**ENSG00000185885**	**IFITM1**	**1.304828318**	**0.003807034**
**ENSG00000137965**	**IFI44**	**1.292143094**	**6.64E-10**
**ENSG00000115267**	**IFIH1**	**1.147805314**	**0.001221134**
*ENSG00000126458*	*RRAS*	*−0.222524777*	*0.035308923*
*ENSG00000175274*	*TP53I11*	*−0.228925103*	*0.038692229*
*ENSG00000168159*	*RNF187*	*−0.235492533*	*0.044616825*
*ENSG00000167244*	*IGF2*	*−0.247562395*	*0.026513466*
*ENSG00000182809*	*CRIP2*	*−0.249868373*	*0.010900659*
*ENSG00000183087*	*GA56*	*−0.268869012*	*0.007495434*
*ENSG00000163017*	*ACTG2*	*−0.332161513*	*0.029633892*
*ENSG00000070404*	*FSTL3*	*−0.344548454*	*0.005595595*
*ENSG00000049540*	*ELN*	*−0.359844309*	*0.021121557*
*ENSG00000130176*	*CNN1*	*−0.410923384*	*0.007865768*

Log2fold change is the average from all statistical tests. Adjusted p-value is reported from EdgeR analysis.

### The Effect of PA and D-glucose Combination Treatment on Müller Cell Gene Expression

**Figure 3:**
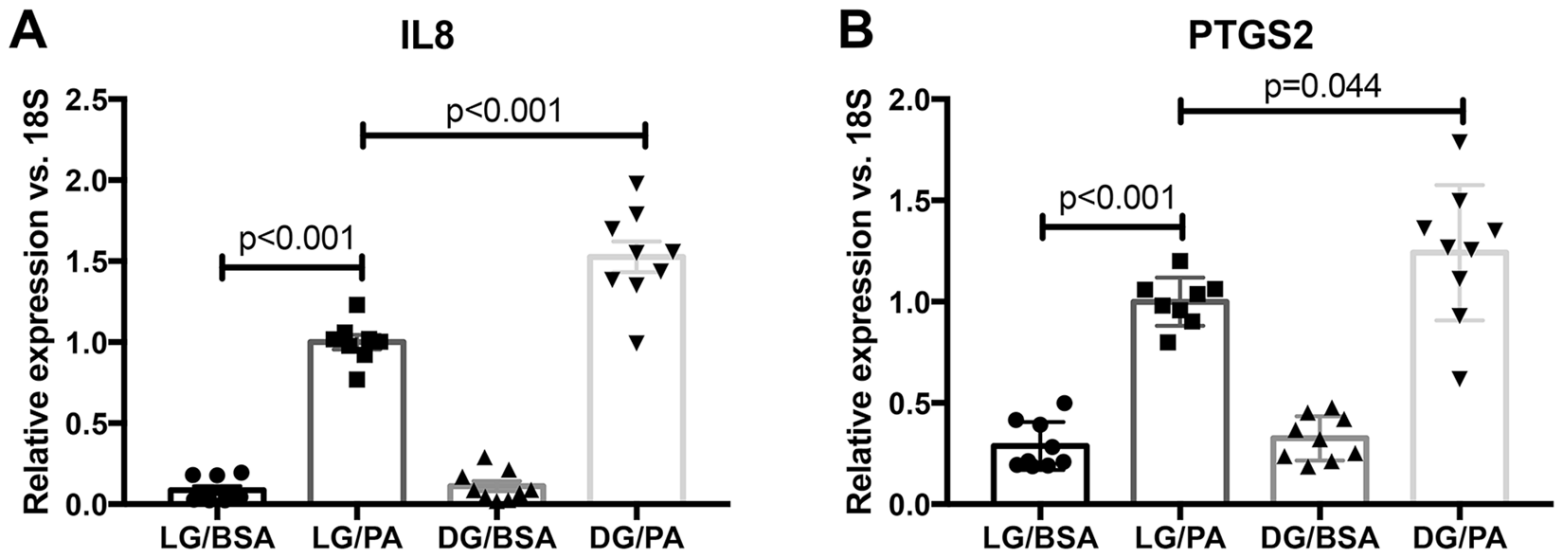
Validation of targets amplified in the DG/PA treatment group. Cells were acquired from 3 different donors, and treated the same as reported for the RNAseq analysis. (**A**) *IL8* and (**B**) *PTGS2* were induced by PA, and further amplified with pre-treatment of D-glucose. Treatments are as follows: LG/BSA (24 hours of L-glucose, followed by 24 hours of L-glucose plus BSA); LG/PA (24 hours of L-glucose, followed by 24 hours of L-glucose plus PA); DG/BSA (24 hours of D-glucose, followed by 24 hours of D-glucose plus BSA); DG/PA (24 hours of D-glucose, followed by 24 hours of D-glucose plus PA). Data is reported as mean ± SEM (n = 9).

We next compared the effect of PA on Müller cells after pre-treatment with D-glucose (DG/BSA vs. DG/PA). While only 9 genes were significantly altered according to all three statistical tests, we used the 51 genes that were altered in any two statistical tests for analysis. In Table 4, the top 10 up-regulated and down-regulated genes are listed. Seventeen genes were up-regulated, and six of those were recognized by KEGG analysis. The only significantly enriched pathway was “Pathways in cancer” (5 hits; p = 8.1E-5) and within this pathway, genes specifically related to proliferation and angiogenesis were enriched. These included genes that encode: Cyclin D1, COX-2, MMP1, IL-8, and c-Jun. Because of their known relationships to diabetes pathology, we focused our validation on *CXCL8* (*IL8*) and *PTGS2*. In Fig. 3, we demonstrate PA induction of these targets, as well as a greater induction by pre-treatment with D-glucose. This finding was also validated for COX-2 protein (Supplementary Fig. 4). PA treatment (LG/BSA vs. LG/PA) induced *IL8* expression by 11.5-fold (p < 0.0001) and *PTGS2* expression by 3.5-fold (p < 0.0001). Compared to PA-treated human Müller cells (LG/PA), D-glucose pre-treated cells (DG/PA) further induced *IL8* expression by 52.6% (p < 0.0001) and *PTGS2* expression by 24.2% (p = 0.0440). The comparison of relative expression determined from qRT-PCR analysis to RNAseq analysis is shown in Supplementary Fig. 3.

**Table 4:** List of top 10 up-regulated genes (bold) and down-regulated (italic) genes in comparison of LG/PA vs. DG/PA samples.

Ensembl Gene ID	Gene Target	Log2fold change	Adj p value
**ENSG00000039600**	**SOX30**	**2.205032696**	**0.008276689**
**ENSG00000159167**	**STC1**	**0.776414785**	**8.46E-07**
**ENSG00000196611**	**MMP1**	**0.702045263**	**0.00040217**
**ENSG00000169429**	**CXCL8**	**0.592836812**	**3.57E-07**
**ENSG00000073756**	**PTGS2**	**0.528668486**	**1.66E-06**
**ENSG00000122861**	**PLAU**	**0.421038355**	**0.004849631**
**ENSG00000162772**	**ATF3**	**0.227905481**	**0.000343642**
**ENSG00000128590**	**DNAJB9**	**0.226338735**	**0.000903843**
**ENSG00000100934**	**SEC. 23A**	**0.221898127**	**0.046332084**
**ENSG00000137831**	**UACA**	**0.195567228**	**0.000562227**
*ENSG00000135480*	*KRT7*	*−0.386967885*	*0.033509088*
*ENSG00000128510*	*CPA4*	*−0.418677262*	*6.12E-05*
*ENSG00000171992*	*SYNPO*	*−0.427503511*	*0.000329537*
*ENSG00000130176*	*CNN1*	*−0.455594004*	*5.73E-05*
*ENSG00000049540*	*ELN*	*−0.460297597*	*0.000196689*
*ENSG00000163431*	*LMOD1*	*−0.462317808*	*4.04E-07*
*ENSG00000137124*	*ALDH1B1*	*−0.464431223*	*8.43E-07*
*ENSG00000163017*	*ACTG2*	*−0.481167848*	*5.73E-05*
*ENSG00000107796*	*ACTA2*	*−0.558187261*	*0.013391793*
*ENSG00000149596*	*JPH2*	*−0.618904013*	*0.007443867*

Bold/italicized genes indicate that these transcripts were also significantly altered in LG/BSA vs. LG/PA sample comparison. Log2fold change is the average from all statistical tests. Adjusted p-value is reported from EdgeR analysis.

## Discussion

The most important finding from this study is that elevated glucose, interacting with the saturated fatty acid, PA, generates a more robust phenotype than either stimulus alone. While the combination treatment yielded relatively small percent increases (i.e. 24–52%), it is important to consider that these inductions are occurring in addition to the stimulated state induced by palmitic acid alone. For example, a 52.6% increase of *IL8* expression following combination treatment with glucose and PA represents a 34-fold induction of gene expression compared to baseline, because PA treatment alone stimulated IL8 expression 22-fold. The observed amplification by D-glucose was specific to PA treatment, as co-treatment with unsaturated fatty acids and D-glucose did not amplify inflammatory transcripts.

Notably, under the experimental conditions in this study, glucose alone had virtually no effect on primary human Müller cells, despite it being the most commonly used stimulus for DR basic research. Yet, the presented results are important for the interpretation of several clinical studies. First, our findings support the results of the FIELD and ACCORD studies, which demonstrated that modulation of dyslipidemia, independent of glucose control, reduced the progression of DR^7,8^. Furthermore, our results suggest that the interpretation of the findings in the DCCT and UKPDS trials, in which glycemic control was significantly associated with DR progression^1,2^, be considered in a broader context because several metabolic changes are associated with glycemic control. Our lab and others have observed no effect of elevated glucose, outside of the effect caused by increased osmolarity of the growth medium^13^, on cellular responses related to inflammation, basement membrane thickening, and angiogenesis in culture^14,17^. Thus, it is worth considering the entire metabolic implication of altering HbA1c and how that might be translated into cell culture systems. Intensive glycemic control is achieved by insulin treatment, and several metabolic changes occur with insulin therapy in diabetics^20^. Elevated circulating lipid is just one of many insults associated with altered insulin signaling. The majority of metabolic alterations have yet to be assessed as stimulators of DR-related pathology. Thus, determining the effect of non-glucose, diabetes-relevant stimuli should remain a high priority for understanding DR onset. The data in the present study, in which conventional elevated glucose conditions yielded no response from Müller cells, demonstrate the need for a more careful consideration of metabolic changes in DR basic research, beyond glucose alone.

Several disease relevant targets were elevated following treatment of human Müller cells with PA alone, while no notable targets were detected with D-glucose treatment alone. Of the most highly expressed genes stimulated by PA, three are from the C-X-C family of chemokines (CXCL2, CXCL3, and CXCL8). These chemokines all signal through the CXCR2 receptor, while CXCL8 also signals through the CXCR1 receptor. These ligands have been implicated in neutrophil migration and chemotaxis^21^. While virtually nothing is known regarding CXCL2 and CXCL3 in diabetic retinopathy, CXCL8 (also called IL-8) has consistently been found to be elevated in the vitreous of diabetic retinopathy patients^22–24^. Other interesting targets on this list (Table 2) include PLIN2 and ANGPTL4, which are well-characterized targets of PPAR-β/δ signaling. This signaling pathway was also significantly enriched with PA treatment (Fig. 1). Our lab has demonstrated that PPAR-β/δ signaling is involved in several pathological steps of DR, including TNFα-induced retinal vascular inflammation, VEGF-induced vascular permeability, and retinal angiogenesis^25–27^. Furthermore, ANGPTL4 has recently been demonstrated to be an important factor in diabetes-induced angiogenesis in PDR^28,29^. These data further demonstrate that PA is a useful stimulus for understanding elements of DR pathogenesis in retinal cell culture, because PA-stimulated cultures faithfully recapitulate important pathogenic endpoints observed in the diabetic retina.

In PA treated cells, we also observed significant enrichment of the neurotrophin pathway. Of the identified targets, nerve growth factor (NGF) was the only ligand up-regulated in this pathway. Notably, NGF is increased in the diabetic rat retina and in tears of diabetic patients^30–32^. While NGF is predominantly involved in neuronal cell survival, it is expressed as proNGF and requires cleavage to exert its pro-survival activity^31^. ProNGF signals through a separate receptor, p75^NTR^, which has been found to cause neuronal cell death and vascular permeability in the retina^33^. In diabetic retinopathy, the ratio of proNGF/NGF is increased, suggesting a greater pro-inflammatory and pro-apoptotic activity^31^. While further work should be directed at understanding the relative amount of proNGF and NGF in PA-treated Müller cell cultures, our data recapitulate the up-regulation of NGF transcription that is observed in DR models and patients.

It is important to consider the relevance of the altered transcripts in this study to changes observed *in vivo*. The analysis of PA-treated Müller cells showed overlap with targets identified by whole transcriptome analysis of STZ-treated animals. Transcripts in several pathways, including angiogenesis (Vegfa, Vegfb and Angptl4), WNT signaling (Sfrp1, Cldn1, Bmp4, Wisp1, and Wnt2b), and inflammation (Ltbp1, Islr, and Bmp4), were elevated in both our PA-treated Müller cell cultures as well as retinas of STZ-treated animals^34^. Additionally, the levels of these transcripts were sensitive to treatment with a p38 MAPK inhibitor, PHA666859, in STZ animals^34^. MAPK signaling was identified as a significantly modulated pathway by both KEGG analysis (Fig. 1) and Biocarta analysis (Fig. 2) in our PA-treated Müller cell samples. Thus, modulation of MAPK signaling may be particularly efficacious for DR. Furthermore, this validates the use of PA-treated Müller cells as a novel platform for high-throughput analysis of therapeutic agents, before going to more expensive pre-clinical animal models.

Compared to treatment with PA alone, PA co-treatment with D-glucose further stimulated a limited subset of targets, predominantly involved in inflammation and angiogenesis. Two of these targets, *IL8* (or *CXCL8*) and *PTGS2*, were verified to be induced by PA and further amplified by co-treatment with D-glucose (Fig. 3). COX-2 (*PTGS2* product) is an inducible enzyme involved in prostaglandin synthesis, and is a target of non-steroidal anti-inflammatory drugs (NSAIDs). NSAIDs and COX-2 inhibitors have been shown to reduce retinal leukostasis and vascular permeability in diabetic rodents, independent of VEGF levels^35^. COX-2 may also be involved in the late stages of disease, because its inhibition prevents the development of ischemia-induced neovascularization in oxygen-induced retinopathy models^36,37^. Additionally, PGE_2_, a downstream product of COX-2 metabolism, is up-regulated in the vitreous of diabetic patients^38^. Our lab has previously shown that PGE_2_ can signal via the EP4 receptor to stimulate retinal angiogenesis^39^. Therefore, our data further validates COX-2 as a potential drug target. More importantly, our data demonstrated the utility of combination treatment of PA and D-glucose for understanding DR pathology in Müller cells.

Interestingly, we identified several angiogenic factors up-regulated by PA and/or DG/PA treatment, including but not limited to: CXCL8, ANGPTL4, COX-2, and VEGF. Notably, these have all been shown to be hypoxia-inducible factors, and in fact, “hypoxia and p53 in the cardiovascular system” was identified by Biocarta analysis as an enriched pathway (Fig. 2). Yet, we demonstrate the induction of these factors in the absence of hypoxia. This suggests that metabolic changes independent of growth factor induction by hypoxia may mediate the progression from early to late angiogenic stages of DR. This finding is supported by a recent study, which demonstrated that altered lipid and glucose usage in Vldlr^−/−^ animals drives retinal *Vegfa* expression via HIF-1α stabilization and subsequent neovascularization in the absence of hypoxia^40^. VEGF up-regulation was observed in photoreceptors exposed to conditions of abundant fatty acids, such as excess palmitic acid in culture^40^. The present study suggests that excessive fatty acids may also affect other highly metabolic cell types in the retina, such as Müller cells.

Modeling a chronic, multifaceted disease like DR *in vitro* remains a challenge, but it is necessary to continue to develop *in vitro* models for several reasons. First, PDR cannot be recapitulated in any known animal models. In rodent models, the disease pathology is mild and never progresses to late stages, so these models lack the potential to yield information about an important aspect of DR – the transition from NPDR to PDR. Second, the use of human primary cells is an important, clinically-relevant complement to *in vivo* animal models for understanding mechanisms of DR pathogenesis. For example, IL-8 is consistently elevated in the vitreous of diabetic patients, yet mice do not express IL-8. While rodents do express functional analogs, they might be regulated differently than IL-8. Therefore, human cells, which produce IL-8, may be important for understanding the mechanism of its induction as well its functional effects in DR pathogenesis. Third, cell culture models that recapitulate disease processes allow for better mechanistic understanding of the disease. However, this relies on the use of appropriate culture conditions and cell types. Virtually all studies assessing glucose-induced behaviors in the literature use either transformed cell lines (rMC-1 or MIO-MI) or rodent-derived Müller cells^41–46^. However, we demonstrated in the present study that elevated glucose did not evoke the same responses from these cells as it did from our human primary cultures. There are several notable differences between primary and transformed Müller cells. For example, our lab has observed that constitutive VEGF production by the human transformed cell line, MIO-MI, is similar to hypoxia-induced VEGF expression levels in primary human Müller cells, suggesting that these cells are not appropriate for experiments of this type. However, it is important to note that these acute cultures likely did not generate advanced glycation endproducts (AGEs), and AGEs may activate Müller cell cultures to a greater extent than glucose alone. Lastly, cell culture models allow for high-throughput analysis in controlled treatment conditions. DR takes years to develop in humans, and months to develop in rodents. We have previously attempted longer culture periods in glucose-enriched media (7+ days) and have seen phenotypic alterations in cultures that are consistent with de-differentiation of primary cells. The culture conditions presented in the present manuscript cause transcriptional changes in line with DR in primary retinal cell culture, while maintaining phenotypic characteristics of these cultured cells, as seen in Supplementary Fig. 1. For these reasons, we believe the culture model described in the present study using human Müller cells and appropriate disease-relevant stimuli represents a useful tool for the DR research community.

## Materials and Methods

### Human Müller cell isolation, culture and treatment

All experiments were approved and performed in accordance with guidelines by the Vanderbilt University Medical Center Institutional Biosafety Committee. Primary human retinal Müller Cells were isolated from human donor tissue (NDRI) within 24 hours post-mortem, using a modified protocol developed by Hicks and Courtois^47^. The retina was dissected from the eye cup and dissociated in Dulbecco’s Modified Eagle Medium (DMEM; Life Technologies; Waltham, MA) containing trypsin and collagenase. Following incubation in dissociation medium, cells were cultured in DMEM containing 10% fetal bovine serum (FBS) and 1x antibiotic/antimycotic solution. Passage 5 was used for all experiments.

Müller cells were cultured in 6-well dishes and at 70% confluence, Müller cells were cultured in serum-reduced conditions (2% FBS) for 12 hours before treatment. Cells were then treated in serum-reduced medium containing either elevated D-glucose (30.5 mM; Sigma-Aldrich; St. Louis, MO) or L-glucose (osmotic control; 5.5 mM D-glucose plus 25mM L-glucose; Sigma-Aldrich) for 24 hours. Next, medium was removed and replaced with the same D-glucose or L-glucose treatment in the presence of either BSA-bound palmitic acid (PA; 250 µM; Sigma-Aldrich) or fatty acid-free BSA control (100 mg/ml in PBS; Sigma). The treatment groups are referred to as the following: LG/BSA (L-glucose 24 hours, L-glucose + BSA 24 hours), LG/PA (L-glucose 24 hours, L-glucose + PA 24 hours), DG/BSA (D-glucose 24 hours, D-glucose + BSA 24 hours), or DG/PA (D-glucose 24 hours, D-glucose + PA 24 hours).

### RNA isolation, RNAseq, and analysis

Treated cells were lysed and RNA purified using the RNeasy mini kit (Qiagen; Valencia, CA) according to the manufacturer’s protocol. Total RNA was isolated and submitted to the Vanderbilt Technologies for Advanced Genomics (VANTAGE) core for RNAseq analysis. RNA sample quality was confirmed using the 2100 Bioanalyzer (Agilent Technologies; Santa Clara, CA). All RNA samples had an RNA integrity number > 8.0. Samples were prepared for sequencing using the TruSeq RNA Sample Prep Kit (Illumina; San Diego, CA) to enrich for mRNA and prepare cDNA libraries. Library quality was assessed using the 2100 Bioanalyzer. Sequencing was performed using a single read, 75 bp protocol on the Illumina HiSeq. 3000 (Illumina).

### Alignment and differential expression

The Vanderbilt Technologies for Advanced Genomic Analysis and Research Design (VANGARD) core performed sequence alignment and differential expression analyses. Alignment and gene mapping was performed using QC3^48^. Differential expression was assessed using MultiRankSeq, which uses the results of three statistical algorithms (DESeq, edgeR, and baySeq) to more rigorously determine significantly altered gene transcripts^49^. Briefly, each algorithm generates a rank of differentially expressed genes based on statistical significance of differential read counts. These ranks are summed, generating a list of transcripts with the greatest differential expression by all three algorithms. For analysis, only transcripts with an adjusted p-value < 0.05 determined by any two (L-glucose/BSA vs. D-glucose/BSA and L-glucose/PA vs. D-Glucose/PA) or all three (L-glucose/BSA vs. L-glucose/PA) algorithms were used for analysis.

### Pathway analysis

Pathway enrichment analysis was performed using the Database for Annotation, Visualization and Integrated Discovery (DAVID; v6.7). Lists of significantly up-regulated genes were submitted to the DAVID website and compared to a human reference gene background. The Kyoto Encyclopedia of Genes and Genomes (KEGG) Pathway annotation and Biocarta annotation was used for pathway enrichment analysis. Pathways were considered significantly enriched with p < 0.05.

### Quantitative real time RT-PCR

After treatment, cells were washed twice with cold PBS and total RNA was collected using the RNeasy Mini kit (Qiagen). Total RNA isolated from the culture wells was reverse transcribed using the High-Capacity cDNA Archive Kit (Applied Biosystems; Waltham, MA). Quantitative RT-PCR was performed in duplicate by co-amplification of cDNA (*CXCL8* or *PTGS2*) vs. a normalization control (*18 S*), using gene-specific TaqMan Gene Expression Assays (Applied Biosystems). The delta Ct method was used to determine relative expression of the targeted mRNA normalized to the selected normalization control. All commercial assays were performed according to the manufacturer’s protocol.

### Statistical Analysis

Data from qRT-PCR experiments were analyzed using the Prism software (GraphPad; La Jolla, CA) using a One-Way ANOVA with Tukey’s post hoc analysis. Values of p < 0.05 were considered statistically significant.

### Data availability

Processed RNAseq data files presented and analyzed in this study available upon request from the corresponding author.

## Electronic supplementary material

Supplementary Material
