# Supplementary material for: Palmitic Acid Induces Müller Cell Inflammation that is Potentiated by Co-treatment with Glucose
Source: Sci Rep. 2018 Apr 3;8:5459. doi: 10.1038/s41598-018-23601-1 (PMC5889388; doi:10.1038/s41598-018-23601-1)
Supplement: Supplementary file 1 — Supplementary Material [file 41598_2018_23601_MOESM1_ESM.pdf]

Palmitic Acid Induces Müller Cell Inflammation that is Potentiated by Co-treatment with Glucose

Megan E. Capozzi[1]*, Meredith J. Giblin[2], John S. Penn[1,2,3]

Departments of [1]Molecular Physiology and Biophysics at Vanderbilt University, [2]Cell and Developmental Biology at Vanderbilt University, and [3]Ophthalmology and Visual Sciences at Vanderbilt University Medical Center

*Corresponding Author

megan.e.capozzi@vanderbilt.edu

8009 Medical Center East

1215 21st Avenue South, Nashville, TN 37232-8808, U.S.A.

Supplementary Material

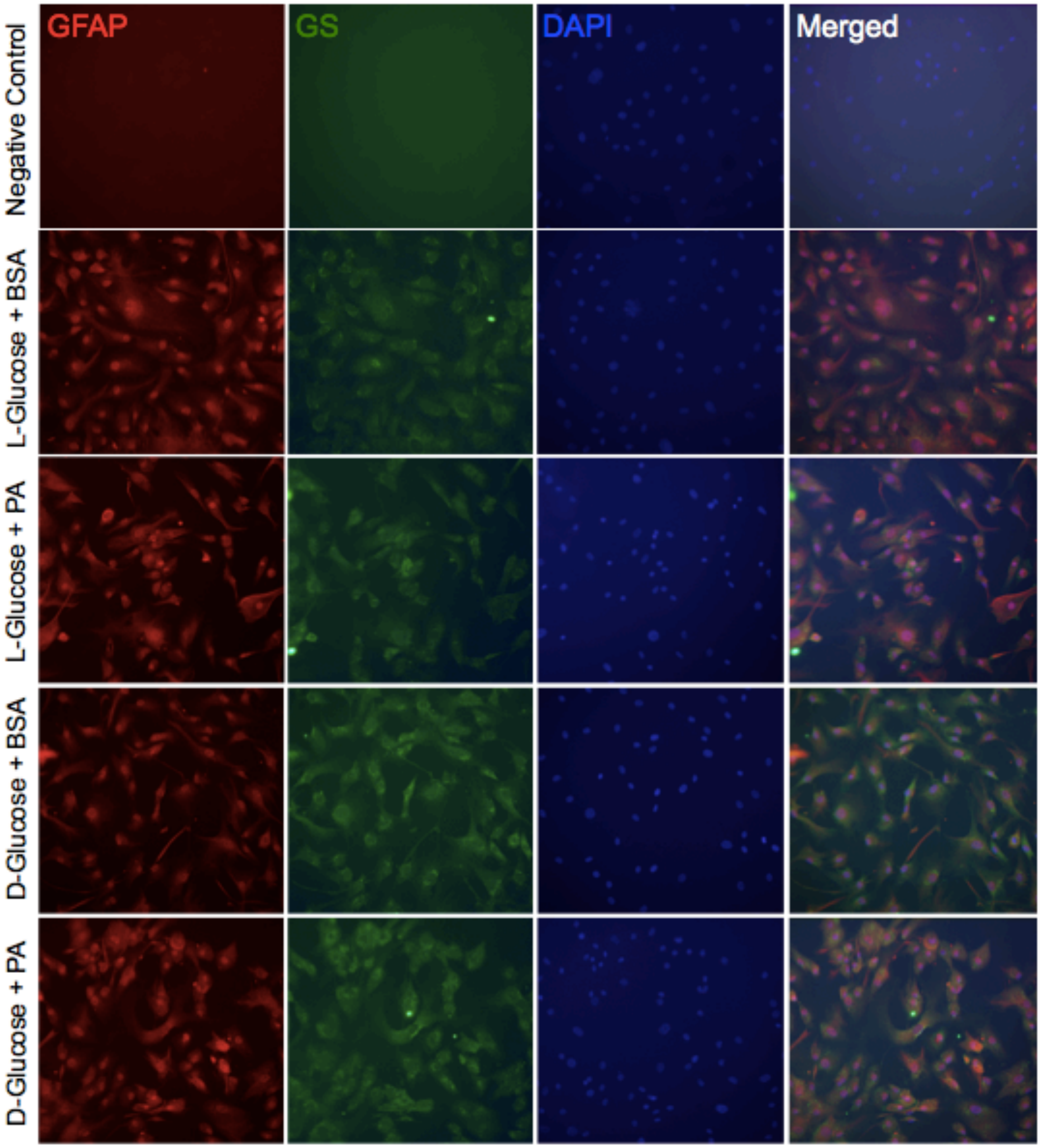

Supplementary Figure 1. Validation of Müller cell phenotype across treatment groups. Müller cells were treated as described for RNAseq analysis. Glial fibrillary activated protein (GFAP) and glutamine synthase (GS) were used for validation of Müller cell phenotype. DAPI was used to identify cell nuclei. All images were taken at 20x magnification.

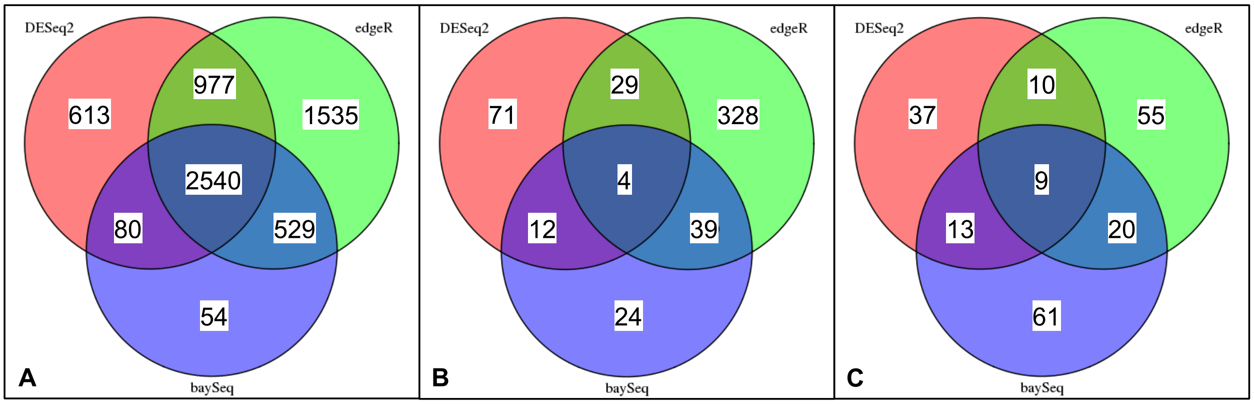

Supplementary Figure 2. Transcripts with adjusted p-value<0.05 for A) LG/BSA vs. LG/PA, B) LG/BSA vs. DG/BSA, C) LG/PA vs. DG/PA.

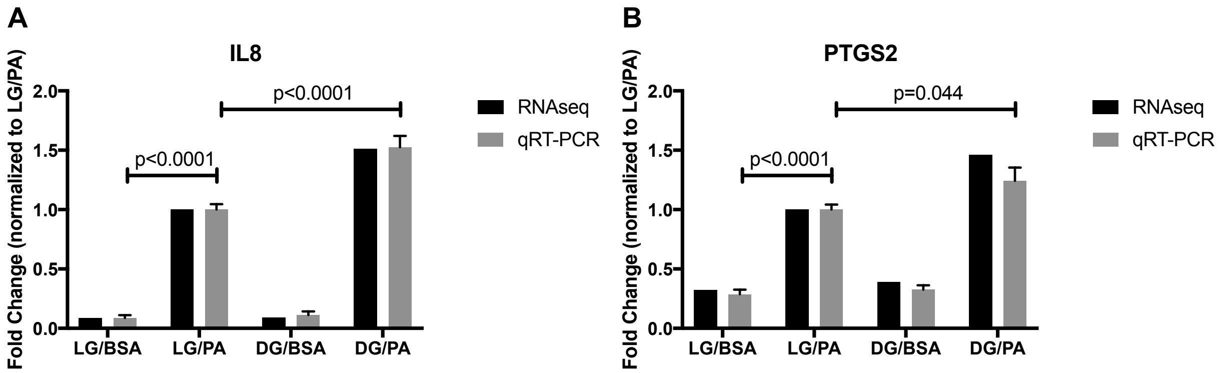

Supplementary Figure 3. Comparison of RNAseq differences to PCR validation expression assays for (A) *IL8* and (B) *PTGS2* expression. Using human Müller cells derived from 3 individual donors, we recapitulated the differential expression observed by RNAseq. The treatment groups are as follows: LG/BSA, 24 hours L-glucose, 24 hours L-glucose + BSA; LG/PA, 24 hours L-glucose, 24 hours L-glucose + PA; DG/BSA, 24 hours D-glucose, 24 hours D-glucose + BSA; DG/PA, 24 hours D-glucose, 24 hours D-glucose + PA. RNAseq bars represent mean value of log2fold change from RNAseq (n=2); qRT-PCR bars represent mean ± SEM (n=9).

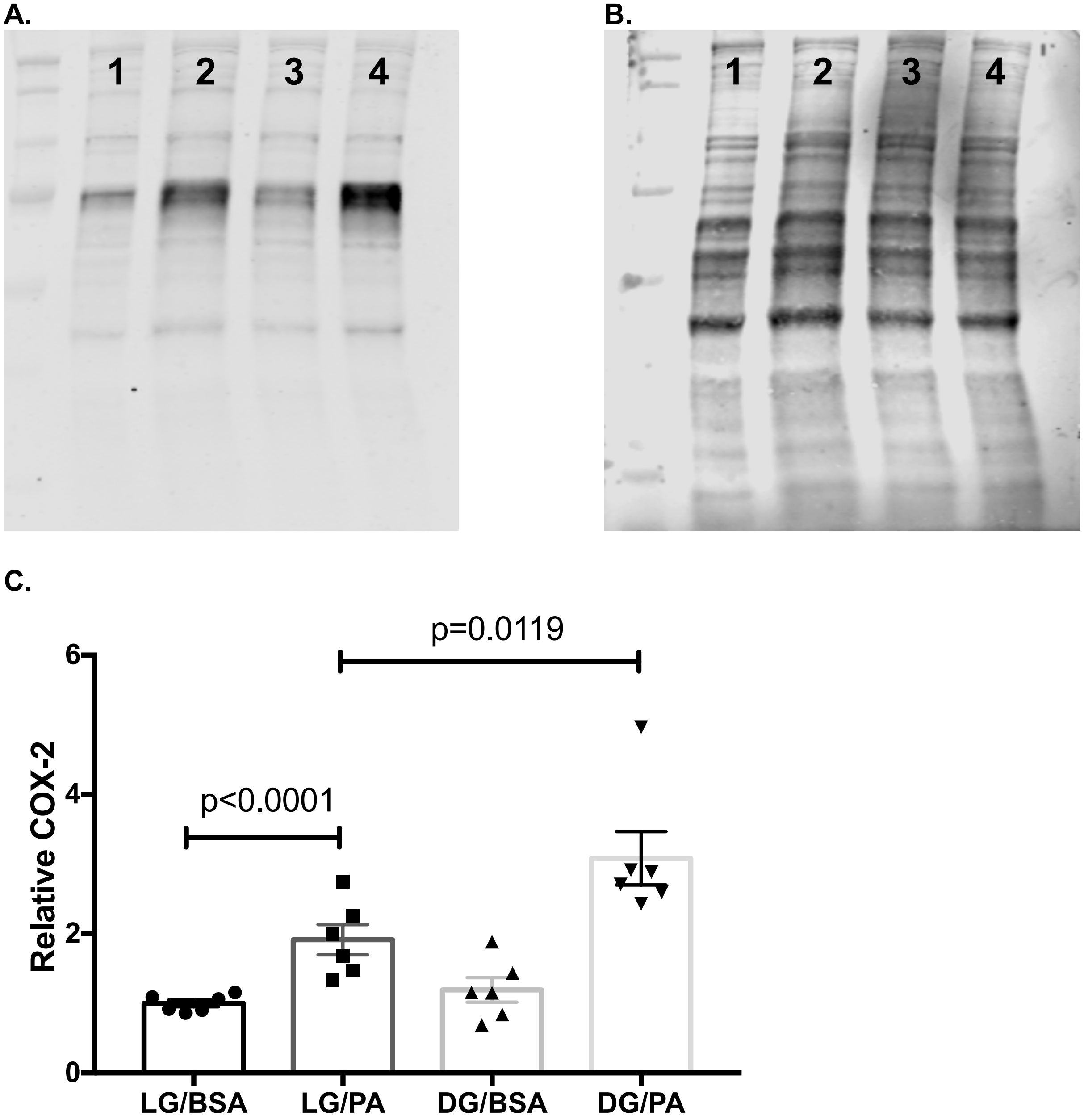

Supplementary Figure 4. Representative blots of (A) COX-2 (immuno-detection at 75kDa) and (B) Total protein, assessed using REVERT total protein stain (LI-COR biosciences; Lincoln, NE). Samples are as follows: 1 – LG/BSA, 2 – LG/PA, 3 – DG/BSA, 4 – DG/PA. (C) Relative quantification of COX-2 protein (normalized to total protein). Bars represent mean ± SEM (n=6).
